# Skating efficiency and technique during roller speed skate using innovative piezoelectric smart socks: an exploratory study

**DOI:** 10.3389/fspor.2025.1554264

**Published:** 2025-07-11

**Authors:** Giulia Bongiorno, Francesco Giuseppe Minisini, Helena Biancuzzi, Francesca Dal Mas, Luca Miceli

**Affiliations:** ^1^ “Friuli Riabilitazione” Rehabilitation Center, Pordenone, Italy; ^2^ Department of Physics, University of Milan, Milan, Italy; ^3^ Department of Economics, Ca’ Foscari University, Venice, Italy; ^4^ Venice School of Management, Ca’ Foscari University, Venice, Italy; ^5^ Collegium Medicum, University of Social Sciences, Lodz, Poland; ^6^ Pain Medicine, IRCCS CRO National Cancer Center of Aviano, Aviano, Italy

**Keywords:** roller skate acceleration, foot pressure distribution, smart socks, roller skate efficiency, educational purpose

## Abstract

This study presents an innovative system for analyzing roller speed skating techniques. It involves socks equipped with piezoelectric sensors and accelerometers, for real-time data collection and synchronization with video signal. Sensors are mixed into cotton fibers, placed in three-foot areas, while the accelerometer is positioned above the external malleolus. Using this system, connected to a mobile APP via Bluetooth, data on foot pressure points, and efficiency metrics, are acquired and calculated. Comparative analysis of a high level competitive and a novice skater highlighted the ability of the system to detect different motor patterns while skating. The high level competitive exhibited different types of strides, with different utilization of foot areas and subsequent accelerometric values. The system could identify the “digital signature” of optimal skating technique, providing training insights for beginners and improving performance tracking. Future applications include building an elite athlete database to refine movement suggestions, with potential adaptability to other cyclic sports.

## Introduction

Roller speed skating requires proper mastery of technique to minimize wheel friction with the ground and increase the athlete's speed during the race (1). Surface electromyographic studies of skaters can provide reference parameters to evaluate the effectiveness of the technical gesture for training purposes, aiming to improve such gestures and prevent injuries (2–4). Modern technologies can assist in developing proper foot placement by analyzing the distribution of forces on the feet during skating through plantar devices (5, 6). Additionally, they can offer a measure of technical efficiency, for example, by analyzing the accelerations generated by the push during skating (7), like practices in other sports such as running (8). However, these technologies have limitations mainly related to equipment costs, specific expertise required for operators, and often lengthy data acquisition times (9). While performing sports activities such inline roller speed skate, the foot and ankle provide both the necessary support and flexibility for weight bearing and shifting. The three areas exposed to high pressures during the linear acceleration phase are the heel, first metatarsal head and hallux, as well described by several authors (10–13). Eils (10) analyzed plantar pressure distribution and stride characteristics in inline skating at two different speeds (18 km/h and 24 km/h). Thirteen advanced male skaters were tested using in-shoe sensors. The results showed that while average force remained similar, faster speeds had significantly higher push-off peak forces and shorter contact times. Pressure distribution patterns were consistent across speeds, with notable peaks in the heel, forefoot, and toes. The findings suggest that higher speed is achieved mainly by increased stride frequency. Inline skating may offer similar physiological benefits to running but with reduced musculoskeletal impact, making it a useful training alternative. Another study of the same author (11) analyzed plantar peak pressure distribution during straight-line inline skating at 18 and 24 km/h in 13 experienced skaters. Using in-shoe pressure sensors, researchers identified three regions of high pressure: the heel, first metatarsal head, and hallux. Increasing speed caused a slight rise in pressure across all regions, especially in the midfoot. However, overall peak pressures remained comparable to walking and lower than those found in running. The study concluded that inline skating generates a distinct pressure pattern and poses minimal overload risk to the foot, making it a potentially joint-friendly endurance activity. In the field of ice skate, similar in various aspects to inline roller skate, authors (12) investigate technical training in speed skating through simulated exercises performed off the ice. Using pedobarography and motion capture systems, four elite skaters were analyzed to assess plantar pressure distribution and phase dynamics during imitation movements. Pressure zones were mapped during four motion phases, highlighting effectiveness and balance, especially in push-off actions. The athlete with the longest experience showed the most consistent pressure patterns. Findings suggest that monitoring pressure distribution can reveal technical readiness and help optimize off-ice training methods, offering valuable insights for coaches aiming to refine skaters’ technique outside traditional ice environments. An ulterior work (13) explores the effectiveness of an off-skate training machine designed to simulate skating mechanics and enhance technique in short track speed skating. A group of athletes trained for 6–8 weeks using the device, which mimicked key movement phases like the push-off and recovery. Performance metrics, including balance, strength, and motion control, were recorded before and after training. Results showed improvements in movement coordination and neuromuscular control, supporting the device's role as a valuable off-ice training tool. The study concludes that targeted, sport-specific dryland training can enhance technical skills and complement on-ice practice. The aim of the research was to use an innovative system based on smart socks equipped with piezoelectric sensors and accelerometers to develop a user-friendly skating analysis system for educational purposes. To do this, the signals deriving from the socks were integrated with a video analysis software and the data obtained were analyzed to develop a reference performance model, derived from a high-level competitive athlete, former world champion, with which to compare the data of a neophyte, paradigm of future users. The innovation brought by these socks is to have the possibility of simultaneously evaluating both the performance aspect, the efficiency of the skating (through the accelerometer parameters that will be described later) and the technical aspect of the same, through the study of the pressure force of the foot inside the skate during the skating task, and to be able to store them for future uses as well as to return to the subject as an evaluation of the performance just performed.

## Methodologies

**Figure 1:**
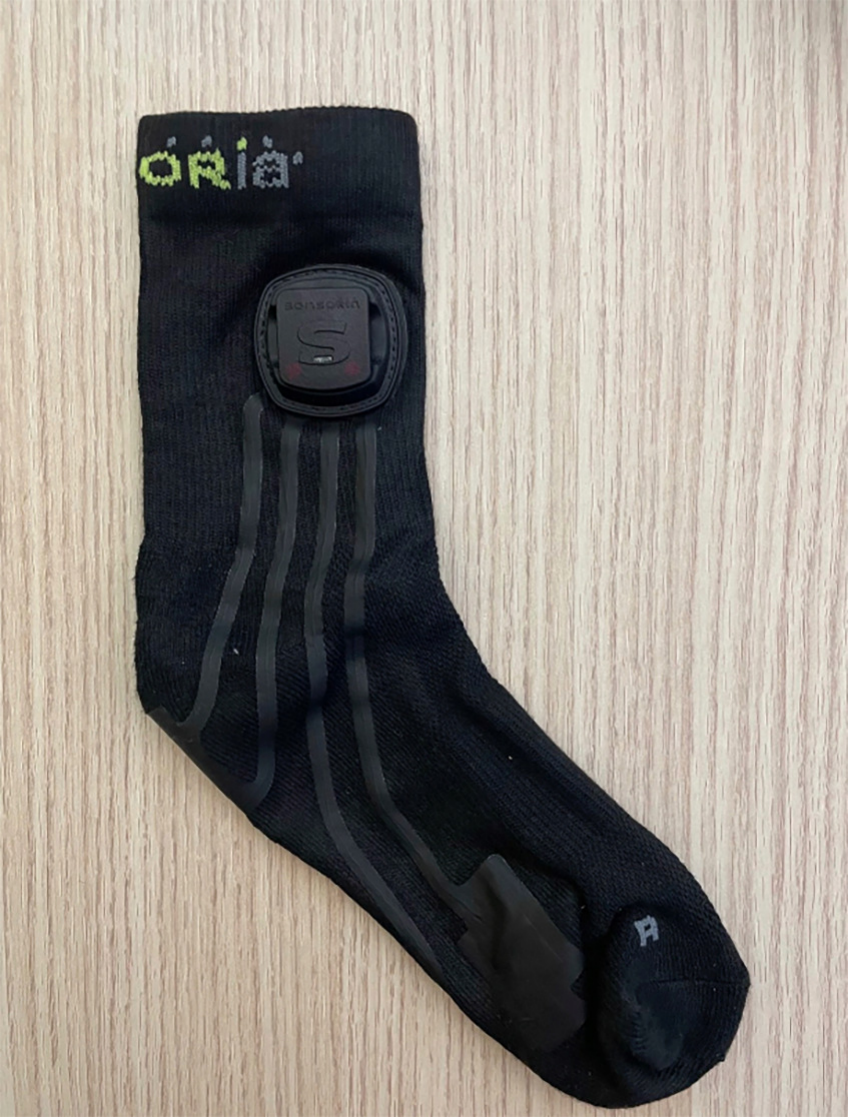
Smart sock with inertial measurement unit (IMU).

**Figure 2:**
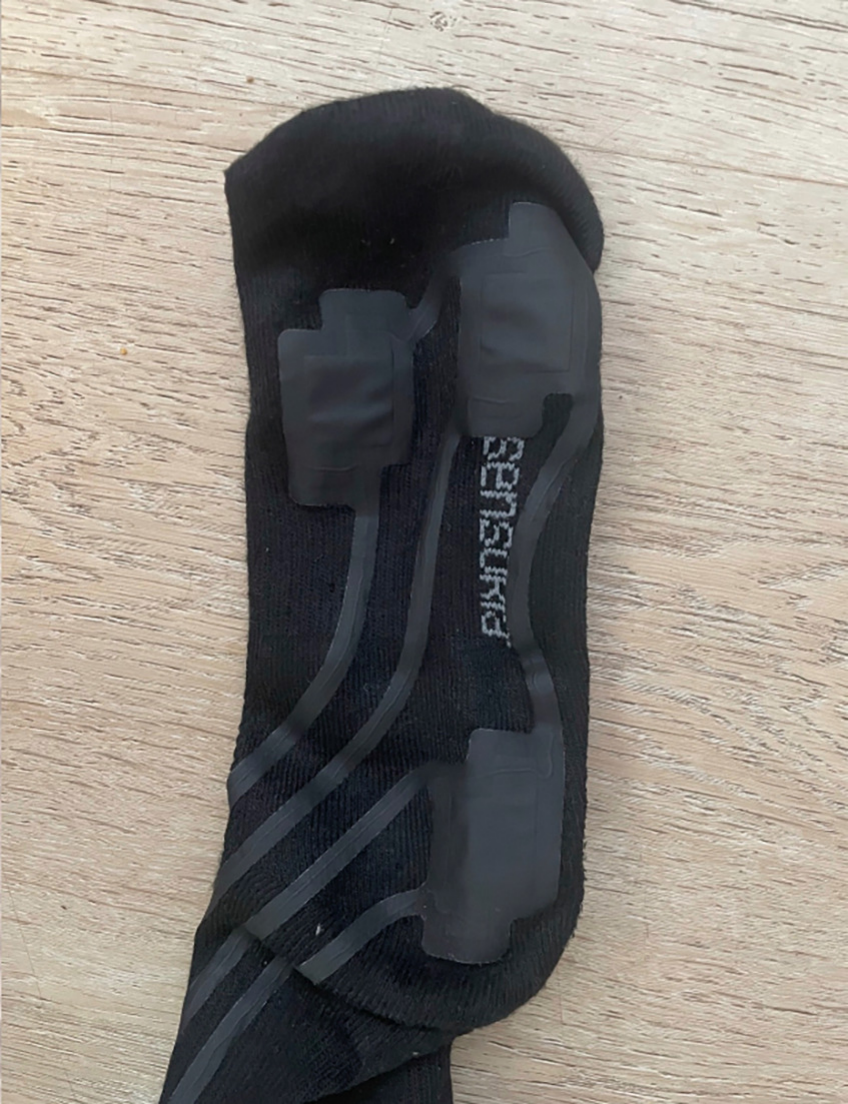
Smart socks with contact areas (smooth surface of the socks).

To test this technology, we engaged a former roller speed skating world champion, already know as performance model in a previous study (4), and a neophyte roller speed skater, with two year experience in this sport, also studied in a previous research (3), to look for any differences in the distribution of forces on the feet and in the ability to generate lateral accelerations during skating. Choice of athletes with different technical maturity was due to the desire to investigate, in this first phase of the project, macro capacity of the hardware and software combination in discriminating differences in the parameters investigated. The tests were performed on a flat, oval, outdoor asphalt track, 200 m in total length, in December 2024, air temperature of about 8 °C, no wind. The speed, corresponding to an average pace of 24 km/h, was measured chronometrically on lap times, knowing the exact length of the track and the time to cover it. Two trials for each participant (and two lap for each trial) was performed by each subject. The purpose of this work was to describe an innovative and user-friendly kit consisting of two socks (Figures 1, 2) equipped with piezoelectric pressure sensors embedded in the textile fibers, specifically at the medial metatarsus, lateral metatarsus, and heel. Each sock is paired with a Bluetooth sensor functioning as an inertial measurement unit (IMU) too. The pressure-sensing surface is sufficiently large (over 2 cm × 1 cm) to prevent artifacts and noise caused by the sock slipping on the foot; acquired signal has always been sufficiently clean and did not require the use of filters (e.g., for very low or very high values), as the results have consistently appeared plausible. Subjects used two different sizes of socks: women (36–40 EU size) and man size (40–44 EU size); sock's thickness was less than 2 mm, so as not to create alterations in the skating. These socks (Sensoria Smart Socks^©^, Redmond, WA 90052, USA) are able of real-time data acquisition of IMU signals across three axes (*X*, *Y* and *Z*), and data from the three previously described piezoelectric sensors, all at frequency of 100 Hz; so, both the piezoelectric and accelerometer systems detected and sent a value to the repository APP every 10 ms.

**Figure 3:**
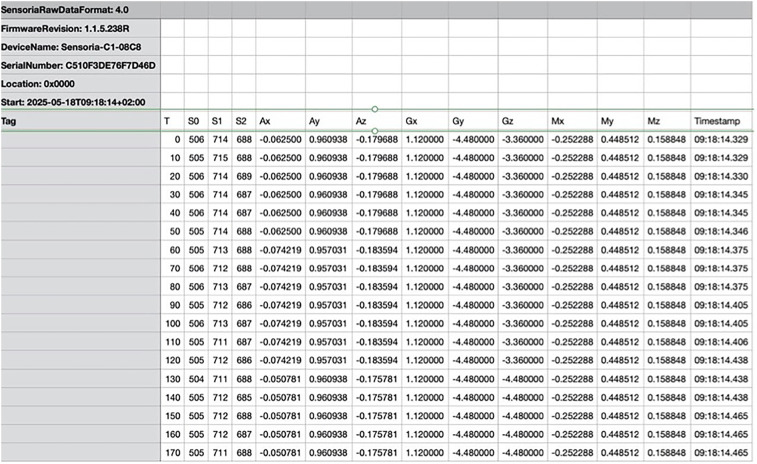
Raw data provided by socks.

The smart socks continuously collect data from the pressure sensors embedded in the fabric. These sensors do not require manual activation; rather, they start recording as soon as the user push the bottom in specific mobile APP. (Sensoria Lab APP^©^). Distinction between walking and skating is not made by the socks themselves, but rather through the subsequent analysis of the collected pressure patterns and timing data. In our study, the identification of movement type (e.g., skating) was based on context and verified by video recordings synchronized with the sensor data. The two sensors, one for each sock, operate independently but have different identification codes, which are indicated both on the sensor itself and in the generated.csv file (Figure 3).

Each file includes time-stamped entries at 100 Hz with columns for:

• Sensor ID (to identify right and left foot)
• Time (ms), sampled every 10 ms
• Pressure values for the three sensors (heel S2, medial S1, lateral S0)
• Accelerometer readings along *X*, *Y*, and *Z* axes (*Ax*, *Ay*, *Az*)
• Gyroscope along *X*, *Y*, and *Z* axes (*Gx*, *Gy*, *Gz*)
• Magnetometer along *X*, *Y* and *Z* axes (*Mx*, *My*, *Mz*)
• Timestamp (h, min, ms)

**Figure 4:**
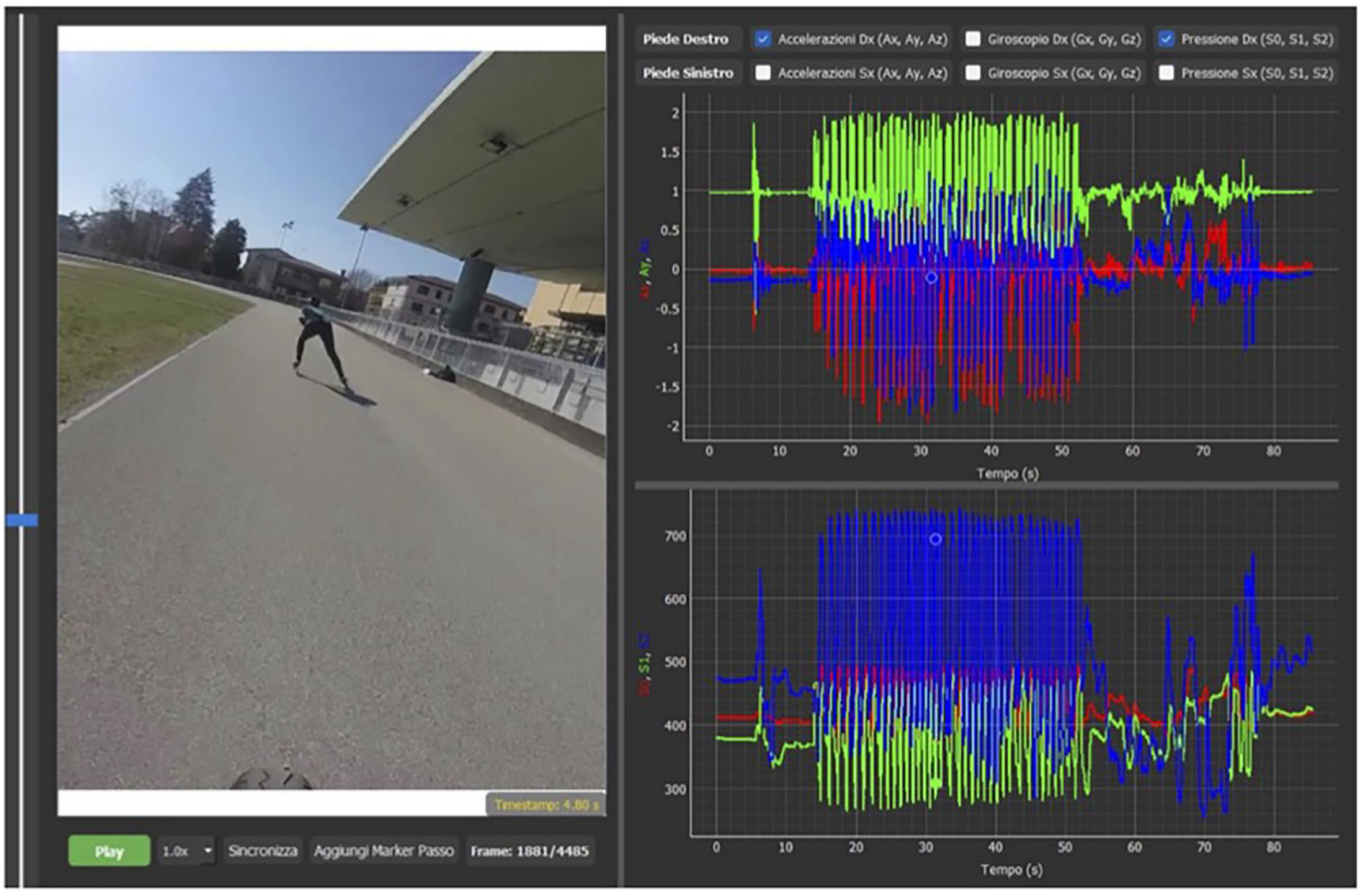
Integration video and sensors data form Sensoria Socks software. Data and image acquired with written consent of the athlete, in accordance with GDPR 2016 law.

This allows us to know which one was positioned on the right side and which on the left. Synchronization between them is achieved through the timestamp (internal clock) of the app itself. The data are then processed separately after synchronization using Microsoft Excel software. The sensors are equipped with rechargeable batteries and can be charged using the dedicated cable and included USB connector. Smart socks are equipped with rechargeable lithium batteries, which guarantee up to 8 continuous hours of data acquisition, as per manufacturer specifications. In our experimental sessions, each trial lasted approximately 10–15 min, and no battery failures or signal interruptions were observed. Additionally, battery status is monitored via the mobile app before and during acquisition, allowing preventive recharge when necessary. Synchronization between the video signal (Figure 4), sampled at 60 Hz, and the data coming from the smart socks (100 Hz) occurs in post-production. The synchronization software allows the video to be displayed on the left side, while on the right side it is possible to view, as desired, all the values derived from the.csv file. In the figure, the values selected are those of the right foot, showing accelerations and pressures at the three points S0, S1, and S2 along the three axes (each represented by a different colored curve) over time, which is shown in seconds on the *x*-axis. On the left side, the exact video timestamp and frame number are also displayed, along with the option to insert markers into the graphs to align the synchronization with the selected marker and video frame and necessary to segment.csv file in “propulsion” and “recovery” phases based on these markers. Each sock sensor is uniquely identified by a hardware ID and a label in the generated.csv file, which allows clear distinction between right and left. The synchronization is performed via the internal timestamp of the app (recorded every 10 ms). Furthermore, manual synchronization is refined using a trigger movement (right and left foot tapping) visible both in the video and in the pressure/acceleration data, aligning the two signals precisely. We process the socks independently after synchronization, enabling specific stride analysis for each foot. An operator analyzes the individual frames and as soon as he detects the beginning of the movement from the video starting from a stand-by situation, he indicates to the software that that frame coincides with the first data source coming from the socks at the beginning of the movement. At that point, the software scrolls the individual frames at a different speed compared to the flow of data coming from the socks in such a way that the last frame also coincides with the end of the subject's last stride. The piezoelectric pressure system is able, starting from a specific baseline value, in the absence of stimulation, specific for each sock and for each of the three sensors (first metatarsal, fifth metatarsal, heel) and determined by its own architecture, to detect, on a scale from 0 to 1,024 points, positive (lowering pressure) and negative (increasing pressure) variations of approximately 500 points values within these limits. The fact that the system does not return absolute pressure values, but relative ones did not create problems in the study, since in this first phase the authors wanted to observe the potential of the instrument and evaluating its general abilities, delegating the ability to express absolute values to a subsequent study and specific calibration. Despite this, the values were indexed on the body weight of the two subjects, to have a beginning of comparison between them. The data derived from the accelerometer, gyroscope and magnetometer were then compared, with results overlapping with those already published with a different acquisition system (sensors from a smartphone) on the same subjects (7). Integration video—data software from Sensoria Socks had the function of synchronizing data obtained by video (external high-definition camera at 60 frame/s) and by socks by dividing skating movement into various phases to better data analyze (e.g., “propulsion” from when the skate touches the ground to when it begins to lift and “recovery” the recovery phase that ends with the beginning of the next “propulsion” phase). Bluetooth acquisition system included a 20-ms delay in sending the raw data unfiltered, and synchronization was done manually. The subject performed a trigger movement (lifting the right skate and placing it back on the ground and then the same task with the left skate) before starting to skate. The start of this movement, analyzed both on the variation of pressures and on vertical accelerations (*x*-axis), allowed an operator, once the acquisitions were completed, to manually synchronize the signals. The fragmentation of the individual skates was then always done manually by the same operator, inserting “cut” signals into the software at the start of each skate (frame in which the skate touches the ground) and now of hemicycle (frame in which the skate leaves the ground, start of the return movement). At this point the software “cut” the csv into many fragments, one for each skate and within them one for the outward movement and one for the return movement. The software was written in “Python” language and is original, developed exclusively for this purpose. The data is then sent to a cloud server in.csv format and has already been used in gait analysis studies (14): in this study, 29 participants completed three walking trials wearing Sensoria Smart Socks while simultaneously walking on the GAITRite mat. The socks showed high accuracy in measuring step count and velocity. Their design enables continuous, wearable gait monitoring, supporting their potential use in both clinical and everyday rehabilitation contexts. Furthermore, specific software was developed to synchronize these signals obtained from smart socks with an external video source, contextualizing the numerical values with frame-by-frame video acquired during the athletes’ skating. The software can segment the previously generated.csv file into sections, each corresponding to a skate stride (further divided into the propulsion and recovery phases, based on the frame where the skate makes and leaves ground contact, respectively) for each foot, placing appropriate markers in the analysis screen. For each skate stride, it is possible to extract duration, foot pressure points within the skate during the propulsion and recovery phases, and calculate efficiency formulas, i.e., the Bongiorno index,—the latero-lateral acceleration percentage respect to the global acceleration expressed by the subject during each stride as a key performance indicator (7)—. Bongiorno index formula (Lateral acceleration (%) = (|*az*|/sqrt (*ax*^2^ + *ay*^2^ + *az*^2^)) × 100), proposed and published by authors but not yet been validated against and motion-capture/force-plate data, was collected and calculated on the *z* axys.

**Figure 5:**
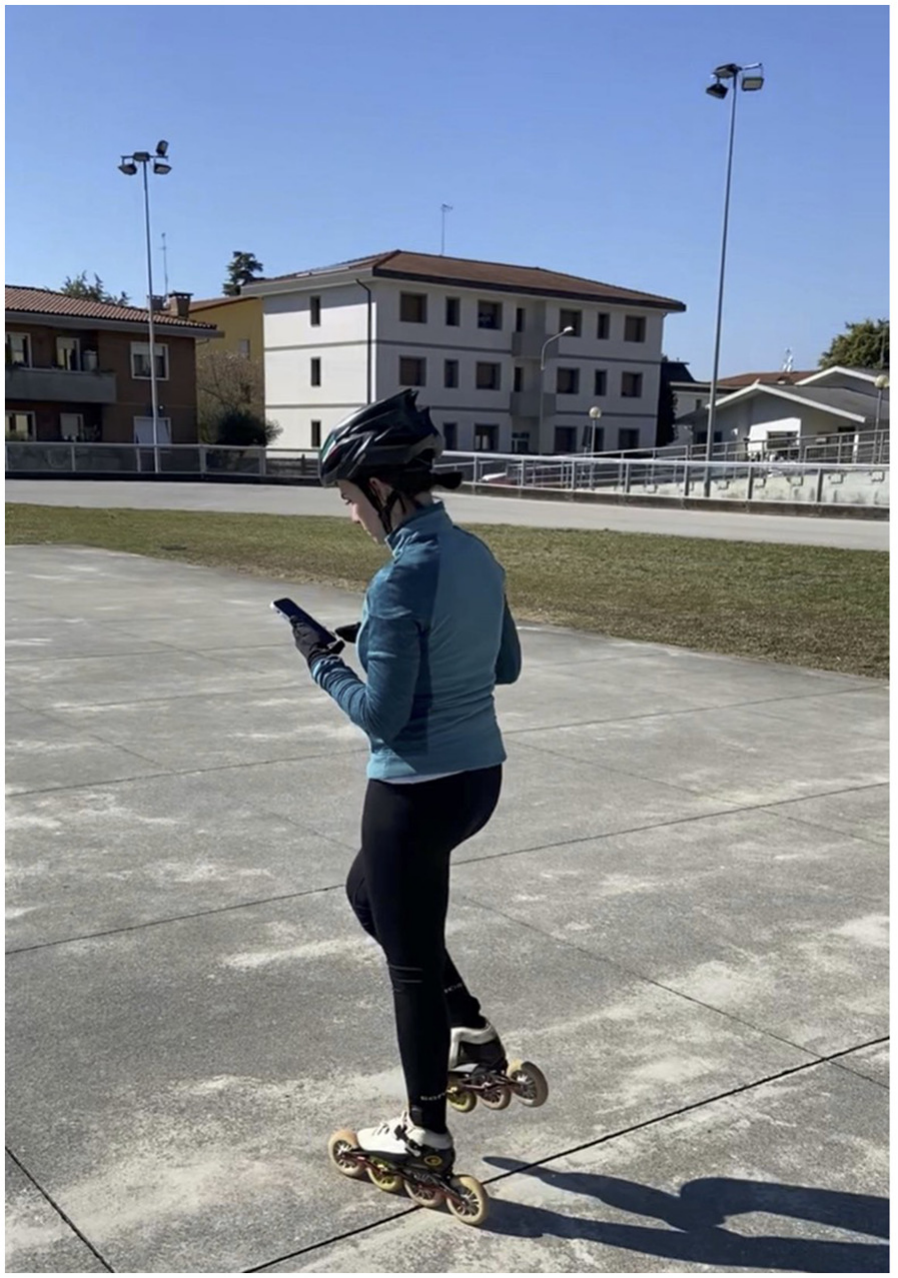
Trigger movement (photo obtained with written permission of depicted athlete).

**Figure 6:**
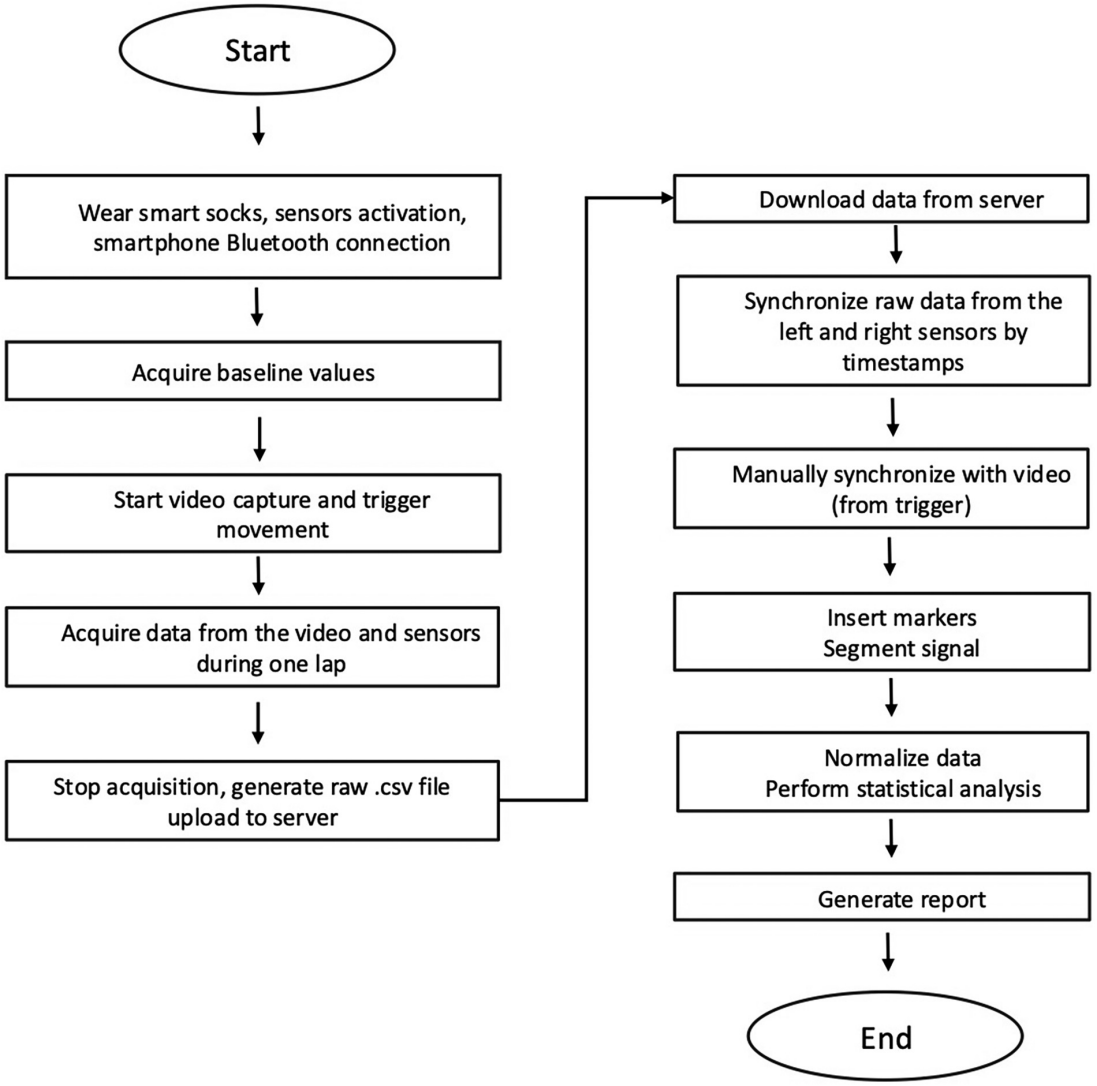
Flow chart of experimental setup and analysis pathway.

“Bongiorno Index” and pressure values differences between feet and between subjects was evaluated. Data were expressed as mean, and the respective confidence intervals 95% were then indicated. Collected data was analyzed with non-parametric Wilcoxon test (left vs. right foot pressure variation and “Bongiorno index” values) and Mann–Whitney test (inter subjects pressure variation and inter subjects “Bongiorno index” evaluation). Significance was obtained with *p* < 0.05. Figures 5, 6 resume experimental setup and processing process pathway.

## Results

**Figure 7:**
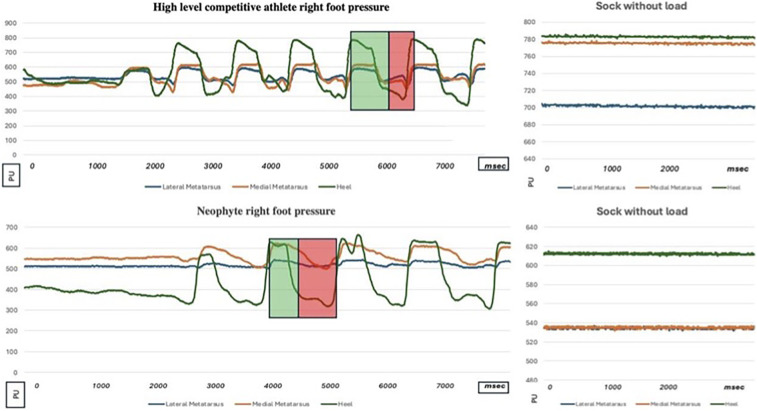
Curves Showing Variations in Pressure Distribution of the Medial Metatarsus, Lateral Metatarsus, and Heel of the Novice Athlete and high-level competitive athlete (former world champion in roller speed skate). In the right side of the figure the stand signal acquisition for both athletes. The skating stride, in this case for the right foot, is divided into a propulsion phase, highlighted by a light green rectangle (during which the “Bongiorno index”—BI—was calculated representing the absolute value of the medio-lateral acceleration relative to the magnitude of the total acceleration), and a recovery phase, highlighted by a light red rectangle. *Y* axis measure units PU: pressure Unit (ranged from 0 to 1,024—see text to major details), *X* axis measure units: ms, milliseconds (1,000 ms = 1 s) *p < 0.05.

**Figure 8:**
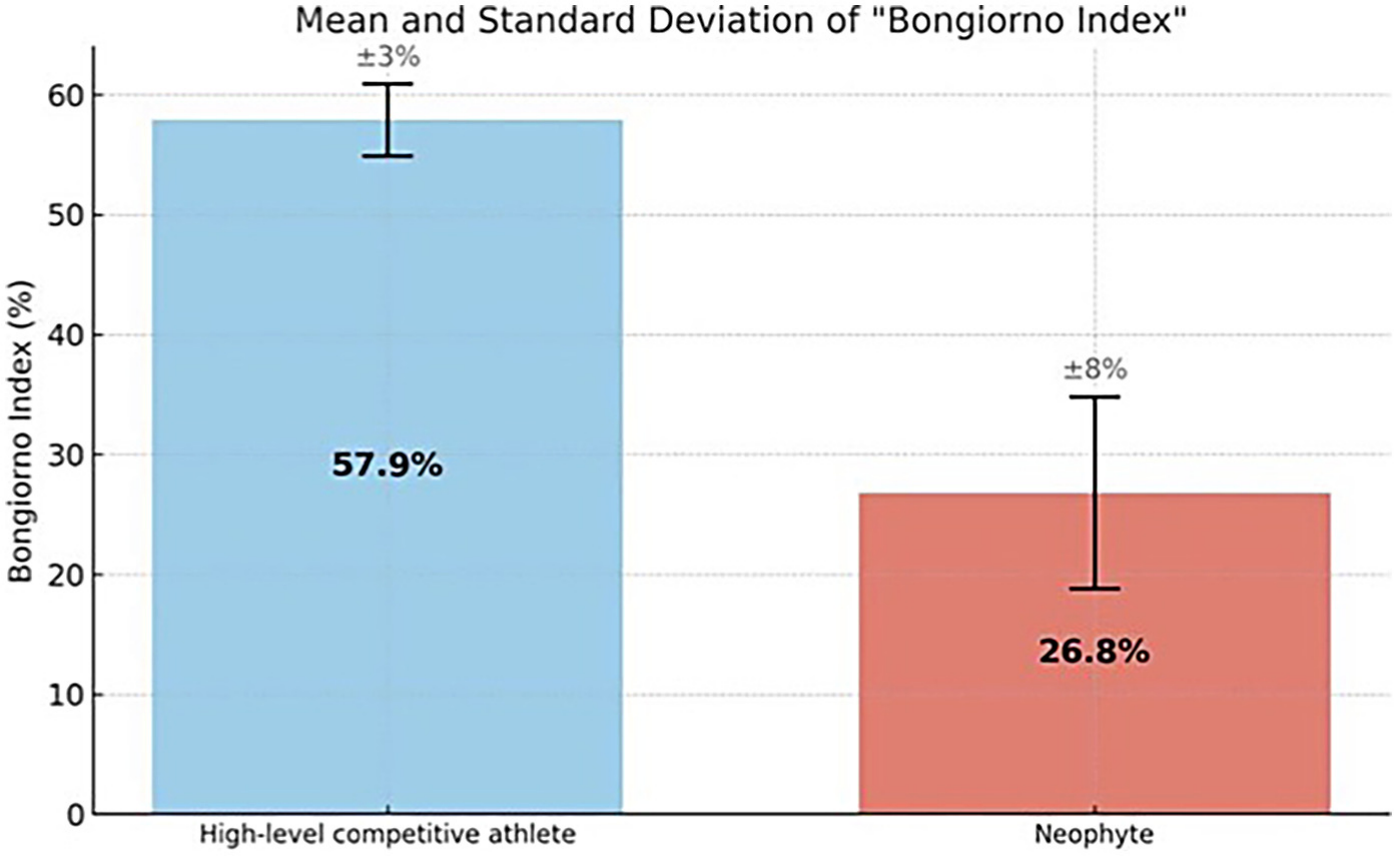
Differences in “Bongiorno Index” (mean and standard deviation) between high level competitive athlete and neophyte.

To evaluate its potential, we preliminary described the stride of a neophyte athlete (two years experienced in roller speed skate) and a high-level competitive athlete former world champion in speed skating, identifying quantitative performance differences (Bongiorno index generated by both subjects) and qualitative technical aspects (foot pressure points during skating), as shown in Figure 7 represent an example of single stride pressure pathway. Subsequent analyses are made considering the mean on five consequent skate per each side (pressure and acceleration indexes). The pushing power, (average variation between the minimum and maximum values detected during skating, a mean of five consequent stride for each side) for high level competitive athlete was 1.6 PU/kg on the lateral foot side (CI 95%: 1.5–1.7), 3.5 in the medial foot side (CI 95%: 3.3–3.6), 6.6 on the heel on the right foot, (CI 95%: 6.4–6.7) 1.8 (CI 95%: 1.6–1.9), 4 (CI 95%: 3.9–4.2) and 6.4 (CI 95%: 6.3–6.6) on the left. Neophyte athlete presented respectively values of 0.4 (CI 95%: 0.2–0.7), 1.5 (CI 95%: 1.1–1.8), and 4 (CI 95%: 3.5–4.5), right side: 0.5 (CI 95%: 0.2–1), 1.3 (CI 95%: 1–1.4), and 4.2 (CI 95%: 3.8–4.5), on the left. Wilcoxon test showed no statistically significative differences from left/right side (*p* = 0.5 high level competitive athlete and *p* = 0.75 athlete neophyte, both with *p* > 0.05). “Bongiorno Index” values (Figure 8) from high level competitive athlete was 57.9% (CI 95%: 56–59), 26.8% (CI 95%: 23–28), in the neophyte athlete in the right side, respectively 60% (CI 95%: 58–61), and 27.5% (CI 95%: 24–28), in the left side. Mann–Whitney test for pressure comparison between two athletes’ pressure showed a value of *p* = 0.04 for left foot, 0.035 for left foot, both statistically significative. An analysis of two “Bongiorno Index” values on five separated strides for each subject, performed with Mann Whitney test for both sides, showed *p* = 0.0079 (*p* < 0.05) for the right side and 0.009 (*p* < 0.05), both statistically significant from high level competitive athlete and neophyte. No significative differences in “Bongiorno index” left/right side for each athlete (*p* > 0.05 at Wilcoxon test).

## Discussion

A brief analysis of the two skaters after single sock calibration (collection of the signal without load on the foot indicated in the right part of the Figure 7 for each athlete, necessary as a preliminary act for each pair of socks) indicates significant differences, both from a quantitative and qualitative point of view. First of all, the high level competitive athlete begins to push on the heel immediately after landing the skate, while the neophyte needs some time to stabilize the foot; then the cyclical nature of the skates is more regular in the high level competitive athlete, with less variability between one and the next strides; the forefoot of the high level competitive athlete also participates more in the push, presenting greater pressure variations than the neophyte from basal values, especially in the lateral part of the foot. Then looking at the numerical results on the pushing power normalized on the athletes’ weight, it can be seen that in the high level competitive the values detected on the lateral, medial and heel forefoot are respectively 1.6–3.5 and 6.6 PU/kg while in the neophyte they are 0.4–1.5 and 4: while maintaining a similar and increasing ratio between the three regions of the foot, the neophyte's values are much lower. They can indicate a minor but not absent technical skill and a lower athletic maturation. The right/left “Bongiorno index” (BI) generated by the two athletes was respectively 57.9/60 (high level competitive) and 26.8/27.5% (neophyte)—Figure 8—, confirming the technical and strength differences between two subjects with difference statistically significant. “Bongiorno Index” was compared absolutely between the two subjects, since it is an index of skating efficiency, or how much of the acceleration generated during skating is used in the lateral part of the skate and is therefore already a proportional value due to a technical skill. The pressure values instead were indexed on the body weight of the subjects to reduce the bias due to the gravitational effect of the body mass on the skate, different between two athletes. Since the novice subject had a body weight of 1.5 times the high level competitive one, one this could have generated the false hypothesis of a good push on the skate from a quantitative point of view, regarding pressure values, where instead the force of gravity too was acting. From these observations, it may be possible in the future identify the “digital signature” of the correct skating movement in speed skating in terms of foot placement.

By “digital signature” we refer to the ability to capture the exact metrics of the skating pattern of a high-level athlete, to be compared with the efficiency that the athlete can produce (the “Bongiorno index”). In the future, this will allow us to identify which variations in skating technique are more or less efficient. Additionally, it could be built a didactic system for novice athletes, allowing the measurement of technical efficiency improvements during training using the described indexes and values. The system proved to be simple, fast, economical, and potentially applicable to other cyclic sports (e.g., running,). Future steps include creating a database of elite male and female athletes at different and controlled speeds. This database could enable an artificial intelligence software to provide suggestions for novices to refine movements and improve their performances. In next phases of the project, we will use supervised learning artificial intelligence to create, with a larger size of subjects, a “virtual coach” for athletes during training sessions. Among the limitations of the work described, it should be noted, in addition to main limitation due to the small sample size (remembering however that the purpose of this work is to propose a new analysis tool and that subsequent studies will be conducted on a larger pooled sample to have precise data to support it), that the world of sport could pose resistance to the use of such advanced technological methods, even more so if they are supported by artificial intelligence, as discussed by other authors (15): these authors examine the stigma surrounding AI chatbots in academic research, addressing concerns about job displacement, data privacy, and academic integrity. It argues that AI chatbots enhance efficiency rather than replace human expertise. The study suggests education, transparency, regulation, and ethical guidelines to foster acceptance and responsible AI integration in research, all highly shareable concepts in our opinion. A positive aspect of this project is to offer the scientific community new performance evaluation tools potentially useful for planning the correct workloads in the world of sport, which are added to the known ones (16) (in example RPE—Rating of Perceived Exertion—, volume-intensity-frequency standardization, and energy expenditure metrics). Smart socks and IMU (inertial measurement units) are therefore an interesting study tool, and they are already object of attention in Literature. A 2018 study (17) compares smart textile systems (STSs) and inertial measurement units (IMUs) for activity monitoring. 18 participants tested a smart undershirt and smart socks vs. a commercial IMU system. STSs were preferred for usability, comfort, and continuous monitoring, while IMUs showed limitations. STSs offer promising potential for wearable sensor applications. A 2020 review (18) explores smart socks and in-shoe systems for foot motion analysis, sports, and medical applications. It compares smart socks (textile-based sensors) and pressure-sensitive insoles (PSI) for gait monitoring, plantar pressure measurement, and injury prevention. As for the filtering, our device returned clean signals without significant noise. Therefore, no filtering was applied in this exploratory phase. However future studies on larger cohorts will consider noise analysis and potential signal preprocessing, especially in more complex outdoor settings. Applications include diabetic foot monitoring, sports performance optimization, and rehabilitation. The authors conclude that smart socks offer better comfort and wearability, while in-shoe systems provide higher accuracy. Specifically, regarding the study of pressures, we can refer to the four previous experiences cited, two on roller skating and two on ice skating. Regarding the first work on roller skating (10), we can note how even in our experience on the high-level athlete, at 24 km/h speed (the same as the comparison work) there is a greater pressure on the medial part of the foot compared to the lateral part, with a similar temporal progression and a peak in the final phase of skating. Similar results on another work (11) on 13 experienced athletes show a lower pressure on the lateral part of the foot, again at 24 km/h. Here we can see how the athletes put greater pressure on the medial/anterior part of the foot compared to the heel compared to the athlete studied by us. This could be due to a difference in skating technique, since the athlete we studied is not only an experienced skater but a former world champion, who could therefore have developed a different support, more oriented on the back of the skate to have greater propulsive force during the push phase of skating compared to the forefoot. Further studies on high-level professional athletes could confirm this first analysis. Reasoning on other kinetic parameters we note how other authors (12) have already been interested in this aspect, even if on ice skating and not roller skating, studying the displacement of the center of mass of a skater during a simulation of movement in the laboratory; we studied the acceleration generated on the track (“Bongiorno index”) and, although we cannot compare the results due to the methodological differences and parameters detected in the two studies, we can hypothesize the importance of focusing on this aspect as well and not only on pressures, something made possible by the devices in our possession in a simple way and in real conditions of use. The same attention, for the research of new technological solutions, always on ice skaters, with the use of a device placed between the shoe and the blade able to detect the pressures generated, pushes us to think that we have used a correct approach to the theme; also in this work we focus not only on the forces but also on other parameters, the displacement of the center of mass of the subject, this time in real conditions during the execution of a curve. Future advancements should improve sensor integration, durability, and real-time data processing for broader medical and athletic applications. This technologic approach has recently been used, with promising results, also for the study of the ankle joint and walking using specific insoles (19, 20). In conclusion, the project was developed with the aim of being able to serve for different sports. In this first phase, roller speed skating was analyzed, but the combined use of socks and video analysis can also be calibrated for example for running patterns. Here the efficiency index maybe will have to be tested and validated on other axes (for example antero-posterior and no longer latero-lateral), while the support and take-off phases of the foot, always identified by single frame video source synchronization with the socks data, will lead to the description of a different performance model, in which the pressure pattern of the various parts of the foot will be normalized on a large cohort of high level competitive athletes and proposed for educational purposes. The optimization of skating technique based on the performance model will be developed by introducing small adjustments to be evaluated in a second study phase by training athletes. The goal is to assess, through the efficiency index (the “Bongiorno index”), whether each modification results in an improvement or not. By exploring multiple attempts—still to be defined—we aim to overcome the main limitation of the current study, namely the inclusion of only a single reference athlete (albeit already a world champion) instead of a broader cohort (which will be the focus of a future study). The technical “compass” guiding this process will be precisely the afore mentioned efficiency parameter.

## Conclusions

In conclusion, the study described—despite the limitation of involving only two subjects—proposes an innovative training method for inline speed skaters, based on the analysis of skating gestures, foot pressure analysis and the extrapolation of skating efficiency, which can be compared against a known performance model. Further studies will be necessary, involving a larger group of high-level athletes, to create a database of movement patterns that can lead to the highest possible level of efficiency in this discipline.

## Data Availability

The original contributions presented in the study are included in the article/Supplementary Material, further inquiries can be directed to the corresponding author.

## References

[1] de Boer RW Vos E Hutter W de Groot G van Ingen Schenau GJ. Physiological and biomechanical comparison of roller skating and speed skating on ice. Eur J Appl Physiol Occup Physiol. (1987) 56(5):562–9. 10.1007/BF00635371 3653098

[2] Bongiorno G Sisti G Dal Mas F Biancuzzi H Varrecchia T Chini G The kinematic and electromyographic analysis of roller skating at different speeds on a treadmill: a case study. Sensors. (2024) 24:5738. 10.3390/s24175738 39275648 PMC11397868

[3] Bongiorno G Sisti G Biancuzzi H Dal Mas F Minisini FG Miceli L. Training in roller speed skating: proposal of surface electromyography and kinematics data for educational purposes in junior and senior athletes. Sensors. (2024) 24:7617. 10.3390/s24237617 39686154 PMC11645072

[4] Bongiorno G Biancuzzi H Dal Mas F Fasano G Miceli L. Roller speed skating kinematics and electromyographic analysis: a methodological approach. Sports. (2022) 10:209. 10.3390/sports10120209 36548506 PMC9781641

[5] Secomb JL Davidson DW Compton HR. Relationships between sprint skating performance and insole plantar forces in national-level hockey athletes. Gait Posture. (2024) 113:436–42. 10.1016/j.gaitpost.2024.07.297 39111226

[6] Wu W-L Hsu H-T Chu I-H Tsai F-H Liang J-M. Selected plantar pressure characteristics associated with the skating performance of national in-line speed skaters. Sports Biomech. (2017) 16:210–9. 10.1080/14763141.2016.1222628 27658963

[7] Bongiorno G Sisti G Tsiotas G Biancuzzi H dal Mas F Miceli L. Artificial intelligence as a potential teaching tool for athletes: when the skate and the smartphone run together. J Sports Med Phys Fitness. (2023) 63(9):974–6. 10.23736/S0022-4707.23.15186-3 37314440

[8] Simoni L Pancani S Vannetti F Macchi C Pasquini G. Relationship between lower limb kinematics and upper trunk acceleration in rec-reational runners. J Healthc Eng. (2020) 2020:8973010. 10.1155/2020/8973010 32015797 PMC6988689

[9] Kaartinen S Venojärvi M Lesch KJ Tikkanen H Vartiainen P Stenroth L. Lower limb muscle activation patterns in ice-hockey skating and associations with skating speed. Sport Biomech. (2024) 23:2233–48. 10.1080/14763141.2021.2014551 34930101

[10] Eils E. Pressure distribution in inline skating straights with different speeds. Paper Presented at the 16th International Symposium on Biomechanics in Sport, Konstanz, Germany (1998). p. 157–60

[11] Eils E Jerosch J. Plantare druckverteilung beim inline skating auf geraden [Plantar pressure distribution in inline skating on straights]. Sportverletz Sportschaden. (2000) 14:134–8. 10.1055/s-2000-8949 11199403

[12] Ma GD. Characteristics of plantar pressure distribution and transmission of double-push technique in female speed wheel slide. J Tianjin Univ Sport. (2007) 22:225–7.

[13] van der Kruk E Reijne MM de Laat B Veeger DHEJ. Push-off forces in elite short-track speed skating. Sports Biomech. (2019) 18:527–38. 10.1080/14763141.2018.1441898 29847206

[14] Yeung J Catolico D Fullmer N Daniel R Lovell R Tang R Evaluating the sensoria smart socks gait monitoring system for rehabilitation outcomes. PM&R. (2019) 11(5):512–21. 10.1002/pmrj.12003 30861329

[15] Dergaa I Fekih-Romdhane F Glenn JM Fessi MS Chamari K Dhahbi W Moving beyond the stigma: understanding and overcoming the resistance to the acceptance and adoption of artificial intelligence chatbots. New Asian J Med. (2023) 1(2):29–36. 10.61838/kman.najm.1.2.4

[16] Dhahbi W Chaabene H Pyne DB Chamari K. Standardizing the quantification of external load across different training modalities: a critical need in sport-science research. Int J Sports Physiol Perform. (2024) 19(11):1173–5. 10.1123/ijspp.2024-0366 39326859

[17] Mokhlespour Esfahani MI Nussbaum MA. Preferred placement and usability of a smart textile system vs. inertial measurement units for activity monitoring. Sensors. (2018) 18:2501. 10.3390/s18082501 30071635 PMC6111998

[18] Drăgulinescu A Drăgulinescu A-M Zincă G Bucur D Feieș V Neagu D-M. Smart socks and in-shoe systems: state-of-the-art for two popular technologies for foot motion analysis, sports, and medical applications. Sensors. (2020) 20:4316. 10.3390/s20154316 32748872 PMC7435916

[19] Davarzani S Saucier D Talegaonkar P Parker E Turner A Middleton C Closing the wearable gap: foot-ankle kinematic modeling via deep learning models based on a smart sock wearable. Wearable Technol. (2023) 4:e4. 10.1017/wtc.2023.3 38487777 PMC10936318

[20] Mun F Choi AJ. Deep learning approach to estimate foot pressure distribution in walking with application for a cost-effective insole system. Neuroeng Rehabil. (2022) 19(1):4. 10.1186/s12984-022-00987-8 35034658 PMC8762884

